# Identification of candidate macrophage-ferroptosis crosstalk genes associated with immune infiltration in myocardial infarction: A bioinformatics analysis

**DOI:** 10.1371/journal.pone.0356358

**Published:** 2026-08-25

**Authors:** Guoqin Wang, Zhulin Zhang, Guanrui Yang, Jing Wang, Teng Ma, Shuang Guo

**Affiliations:** Department of Cardiology, Shanxi Cardiovascular Hospital, Taiyuan, Shanxi, China; Karpagam University: Karpagam Academy of Higher Education, INDIA

## Abstract

Globally, myocardial infarction (MI) remains a major cause of morbidity and mortality. Macrophage-mediated inflammation and ferroptosis-related stress responses have both been implicated in MI; however, transcriptomic identification of macrophage- and ferroptosis-related candidate genes requires rigorous control of false-positive findings. In this revised study, two peripheral blood transcriptomic datasets, GSE29532 and GSE48060, were integrated after probe annotation, independent normalization, merging based on common genes, and ComBat batch correction. Batch-correction quality was evaluated using both visualization and quantitative metrics. Differential expression analysis was performed using the limma package with adj.P.Val < 0.05 as the primary threshold. A total of 213 differentially expressed genes were identified, including 101 upregulated and 112 downregulated genes. Intersecting FDR-supported DEGs with macrophage-related and ferroptosis-related gene sets identified seven primary MFRDEGs: SMAD7, MMD, PTPN6, DDIT3, AKR1C3, PHF21A, and ACSL1. GO enrichment analysis was interpreted as exploratory functional annotation because of the small input gene set, whereas whole-ranked-gene GSEA highlighted TNFA/NF-kB inflammatory signaling, complement-related innate immune signaling, and reactive oxygen species-related transcriptional programs. Because the revised PPI analysis did not produce reliable interactions among the seven primary MFRDEGs, candidate gene prioritization was performed instead of defining interaction-derived hub genes. ssGSEA suggested a robust neutrophil-related alteration in MI, while macrophage- and monocyte-related signatures showed trend-level changes after FDR correction. Exploratory ROC analysis was performed for selected candidate genes. RT-qPCR validation further showed that SMAD7, PTPN6, DDIT3, PHF21A, and ACSL1 were increased, whereas MMD and AKR1C3 were decreased in AMI peripheral blood samples, consistent with the transcriptomic results. These findings provide FDR-supported candidate-level evidence linking macrophage- and ferroptosis-related transcriptional alterations to immune signatures in MI and warrant further validation in larger cohorts and mechanistic studies.

## 1 Introduction

Myocardial infarction (MI) is a severe life-threatening disease due to sudden coronary artery occlusion. This leads to necrosis of cardiomyocytes because of insufficient blood supply, inducing cardiac dysfunction. Based on recent epidemiological surveys, the global incidence of MI is nearly 65 cases per 100,000 individuals, with a mortality rate of around 30%. MI imposes a significant global burden of morbidity and mortality. MI is a major public health issue, with millions of reported cases documented annually [1,2]. Despite advances in medical treatment and percutaneous coronary intervention (PCI), MI has a poor prognosis associated with high rates of recurrent cardiovascular events and death [3].

Existing studies have established that macrophages play pivotal regulatory roles in post-MI inflammatory responses, clearance of necrotic tissue, and subsequent cardiac repair [4]. After myocardial injury, macrophage-related immune responses may participate in inflammatory amplification, efferocytosis, extracellular matrix remodeling, and the transition from tissue injury to repair [4]. Although peripheral blood transcriptomes cannot directly represent the infarcted myocardial microenvironment, they may reflect systemic immune activation and provide accessible information for identifying candidate genes associated with MI-related immune remodeling.

Concurrently, ferroptosis has been increasingly recognized as a key contributor to myocardial injury and adverse ventricular remodeling following acute MI [5]. Ferroptosis is closely associated with lipid peroxidation, oxidative stress, and iron-dependent cell death, which may intersect with inflammatory signaling and macrophage-related immune regulation during MI progression [5]. Therefore, genes related to both macrophage biology and ferroptosis may provide candidate molecular clues linking immune inflammation, stress responses, and transcriptional changes related to regulated cell death in MI.

Despite these advances, systematic integrative analyses examining macrophage- and ferroptosis-related transcriptional signals and their associations with immune infiltration in MI remain limited. In addition, integrated analyses based on public microarray datasets require careful control of false-positive findings, especially when datasets from different platforms are combined. To address these issues, we performed an FDR-controlled reanalysis of peripheral blood transcriptomic datasets GSE29532 and GSE48060 to identify macrophage- and ferroptosis-related candidate genes, evaluate their associations with immune-cell-related transcriptional signatures, and provide reproducible candidate-level evidence for future validation. The present study should therefore be interpreted as a hypothesis-generating bioinformatics analysis rather than definitive proof of causal mechanisms or clinical diagnostic biomarkers.

## 2 Materials and methods

### 2.1 Ethics approval and consent to participate

The study protocol received ethical approval from the Institutional Review Board of Shanxi Cardiovascular Hospital (Approval No. 2025xxg626). The study was conducted in accordance with the principles of the Declaration of Helsinki. All participants provided written informed consent.

### 2.2 Data collection and gene-set sources

From the Gene Expression Omnibus (GEO) database [6], two datasets (GSE29532 [7] and GSE48060 [8]) were retrieved. The chip platform for GSE29532 was GPL5175, and that of GSE48060 was GPL570. GSE29532 contained 49 MI and 6 Normal control (NC) samples, while GSE48060 contained 31 MI and 21 NC samples. Thus, the integrated analysis included 80 MI and 27 NC samples. All the specific information is presented in Table 1.

**Table 1:** GEO Microarray Chip Information.

	GSE29532	GSE48060
Platform	GPL5175	GPL570
Species	Homo sapiens	Homo sapiens
Tissue	blood samples	blood samples
Samples in MI group	49	31
Samples in NC group	6	21
Reference	PMID: 23535507	PMID: 24801707

Note: GEO, Gene Expression Omnibus; MI, Myocardial Infarction.

The macrophage-related genes (MRGs) were obtained from the GeneCards [9] (https://www.genecards.org/), MSigDB database [10] (https://www.gseamsigdb.org/gsea/msigdb), and peer-reviewed published literature. When retrieving genes from the GeneCards database, “macrophage” was used as the search keyword, protein-coding genes were retained, and entries with a Relevance Score > 1 were retained. Subsequently, the macrophage-associated candidate gene set was generated by merging the obtained results from the MSigDB database and the gene sets curated from published literature, followed by duplicate removal. Moreover, the keywords “macrophage-related” was used to retrive published literature from PubMed [11–13]. The driver, suppressor, marker, and unclassified datasets were downloaded from the FerrDb database [14]. Gene symbols from all gene sets were standardized before downstream intersection and sensitivity analyses.

### 2.3 Probe annotation, normalization, and batch correction

After downloading the raw expression matrices, probes were first converted to gene symbols based on the platform annotation files, and unannotated probes were removed. When multiple probes corresponded to the same gene, their expression values were merged into a single gene-level expression value according to preset rules, and the gene expression matrix was ultimately obtained. Subsequently, each dataset was independently normalized, and the expression matrices were integrated after unified gene annotation for subsequent analysis. After retaining common genes shared by the two platforms, the merged expression matrix contained 15,332 common genes and 107 samples.

Given that both GSE29532 and GSE48060 are expression profile datasets of MI and control samples derived from human peripheral blood, with good comparability in terms of research subjects and sample types, the two datasets were integrated after uniform probe annotation and consolidation into a gene expression matrix. The R package limma [15] was used to normalize the combined datasets. Considering that systematic technical differences may exist between different microarray platforms, the sva package [16] was subsequently used to correct batch effects. In the revised analysis, the dataset/platform was defined as the batch variable, and disease group information was retained in the model matrix using model.matrix(~ group), thereby reducing the risk of removing MI-associated biological variation during batch correction.

Batch-correction performance was evaluated using expression-distribution boxplots, principal component analysis (PCA) [17], density plots, relative log expression plots, variance-explained analysis, and silhouette scores. The mean batch-explained variance decreased from 0.5920 before correction to 0.00176 after correction, and the batch silhouette score decreased from 0.6278 to 0.0184, supporting improved cross-platform integration. kBET/LISI metrics were not calculated because compatible local implementations were unavailable; no values were imputed or fabricated.

### 2.4 FDR-based differential expression analysis

All samples were assigned to the MI and NC groups. Differential expression analysis was performed using the R package limma [15]. To address multiple testing in the high-dimensional transcriptomic dataset, Benjamini-Hochberg-adjusted *P* values were used as the primary statistical criterion. Genes with adj.P.Val < 0.05 were defined as differentially expressed genes (DEGs) in the primary analysis. The previous permissive rule based on nominal *P* values and |logFC| > 0 was not used as the main screening criterion in the revised analysis and was retained only as an exploratory sensitivity reference. The results were visualized using a volcano plot generated with the R package ggplot2.

### 2.5 Identification of FDR-supported primary macrophage and ferroptosis related differentially expressed genes (MFRDEGs)

To identify the MFRDEGs, we intersected the DEGs derived from differential expression analysis of the combined datasets with the MRGs and Ferroptosis Related Genes (FRGs), and visualized the overlapping genes using a Venn diagram. The R package pheatmap was used to generate the heatmap. In the revised workflow, primary MFRDEGs were defined as the intersection of FDR-supported DEGs, MRGs, and FRGs. Based on the primary threshold of adj.P.Val < 0.05, seven FDR-supported primary MFRDEGs were identified: SMAD7, MMD, PTPN6, DDIT3, AKR1C3, PHF21A, and ACSL1. To evaluate the robustness of candidate selection, sensitivity analyses were performed using alternative DEG thresholds and different MRG/FRG gene-set combinations.

### 2.6 Gene ontology (GO) enrichment and gene set enrichment analysis (GSEA)

The GO [18] enrichment analysis of the primary MFRDEGs was conducted using the R package clusterProfiler [19]. GO enrichment includes three dimensions: biological process (BP), cellular component (CC), and molecular function (MF). GO enrichment was performed using clusterProfiler:enrichGO with org.Hs.e.g.,db annotation and Benjamini-Hochberg correction. No custom universe was specified in the final GO enrichment analysis; therefore, the default org.Hs.e.g.,db GO-annotated background was used. Because the input set contained only seven FDR-supported primary MFRDEGs, the GO results were interpreted as exploratory functional annotations rather than robust pathway-level mechanistic evidence. Primary Kyoto Encyclopedia of Genes and Genomes (KEGG) enrichment did not yield stable significant terms and was therefore not emphasized as a main result in the revised manuscript.

GSEA [20] was utilized to evaluate the distribution trend of genes in predefined gene sets within a gene list ranked by correlation with phenotypes, thereby determining their contribution to phenotypes. In this study, GSEA was performed using the full ranked gene list rather than a DEG-truncated gene list. Genes were ranked using the limma moderated t statistic when available. Normalized enrichment score (NES), nominal *P* values, and FDR values were reported. FDR-significant and biologically relevant pathways, including TNFA/NF-kB signaling, Complement, and reactive oxygen species-related signaling, were selected for display.

### 2.7 Candidate prioritization instead of interaction-derived hub-gene definition

The revised STRING/Protein-Protein Interaction (PPI) analysis was performed using STRING [21] to assess whether the FDR-supported primary MFRDEGs formed a reliable interaction network. However, the seven primary MFRDEGs did not yield reliable interactions for defining an interaction-derived central-gene model. Therefore, no definitive PPI-derived hub-gene set was defined in the revised manuscript. Instead, candidate prioritization was based on multiple candidate-level features, including log2FC, adj.P.Val, robustness across sensitivity analyses, dataset-level expression-direction consistency, immune-correlation evidence, exploratory Receiver Operating Characteristic (ROC) performance, and biological relevance. The previous cytoHubba-based hub-gene screening and mRNA-microRNA (miRNA)/mRNA-transcription factor (TF) regulatory-network analyses were not retained as main analyses in the revised manuscript.

### 2.8 Single-sample gene-set enrichment analysis (ssGSEA) immune infiltration analysis

ssGSEA [22] was used to quantify the relative abundance of immune-cell infiltration based on the specific infiltration matrix results of immune cells in MI and NC groups. Subsequently, the R package ggplot2 (Version 3.5.1) was used to generate heatmaps and group comparison plots to illustrate the differences in immune-cell infiltration between the two groups. In the revised analysis, differences in immune-cell-related scores between MI and NC samples were assessed using Wilcoxon rank-sum tests, and *P* values were corrected across immune-cell comparisons using the Benjamini-Hochberg FDR procedure. Furthermore, the correlation heatmap was produced using the R package pheatmap based on correlations between immune cells calculated by the Spearman algorithm.

### 2.9 Candidate gene-immune cell correlation analysis

Associations between candidate gene expression and immune-cell-related ssGSEA scores were evaluated using Spearman correlation analysis. *P* values were adjusted across tested gene-cell pairs using the Benjamini-Hochberg FDR method. Only FDR-significant correlations were emphasized in the main interpretation.

### 2.10 Exploratory ROC analysis

To evaluate the discriminatory performance of candidate genes in distinguishing MI from NC samples, ROC curves were drawn using the R package pROC [23]. Area under the curve (AUC), 95% confidence intervals, and optimal cutoffs were estimated where available. ROC results were interpreted only as exploratory discriminatory performance of candidate genes and were not considered validated diagnostic models or clinical biomarkers.

### 2.11 Collecting peripheral blood from acute myocardial infarction (AMI) patients and healthy controls

Based on the bioinformatics analysis, the peripheral blood samples were collected for reverse transcription quantitative real-time polymerase chain reaction (RT-qPCR) validation, and the expression levels of candidate genes were compared between the MI and the NC groups. This investigation enrolled a total of 12 participants between August 1, 2025, and September 30, 2025, comprising 6 AMI cases and 6 age- and sex-matched healthy controls. AMI subjects were consecutively recruited from the Emergency department of Shanxi Cardiovascular Hospital, with strict adherence to the 2017 ESC STEMI diagnostic guidelines. Inclusion criteria required: (1) characteristic ischemic symptoms; (2) electrocardiographic evidence of ≥ 1 mm ST-segment elevation in ≥ 2 adjacent leads or newly developed left bundle branch block; (3) cardiac biomarker levels exceeding the 99^th^ percentile upper reference limit; and (4) angiographically confirmed coronary artery stenosis/occlusion. We excluded patients presenting with non-st-segment elevation myocardial infarction (NSTEMI) or unstable angina. Peripheral blood specimens (1 mL) consisted of residual blood remaining after routine complete blood count testing obtained from AMI patients within 24 hours of symptom onset and from control participants who were hospital inpatients without coronary heart disease. The study protocol received ethical approval from the Institutional Review Board of Shanxi Cardiovascular Hospital (Approval No. 2025xxg626). The study was conducted in accordance with the principles of the Declaration of Helsinki. All participants provided written informed consent.

### 2.12 RNA isolation and RT-qPCR

Following sample collection, the relative expression levels of candidate genes were quantified by RT-qPCR. In the updated validation experiment, RT-qPCR was performed for the seven FDR-supported primary MFRDEGs: SMAD7, MMD, PTPN6, DDIT3, AKR1C3, PHF21A, and ACSL1. Total RNA was extracted from peripheral blood using TRIzol Reagent (yuan ye, MF034−01). cDNA was synthesized using 2XM5 HiPer SYBR Premix Es Taq (Mei5bio, MF787−01). Gene expression was examined with a Roche480 LightCycler® 96 real-time PCR System using the qPCR SYBR Green Master Mix (Mei5bio, MF787−01). Samples were run in triplicate, and their Ct values were averaged. Relative quantitation was performed using the 2^−△△Ct^ method. GAPDH was used as the reference gene. All qPCR primers used are listed in **S1 Table**.

### 2.13 Statistical analysis

In this study, statistical thresholds were defined independently for each analytical stage-tailored to the specific biological context, analytical objectives, and data characteristics. Differential expression analysis served as the primary filter for candidate gene identification, whereas functional enrichment analysis and GSEA were applied to interrogate coordinated transcriptional shifts at the pathway and gene set levels, respectively. For analyses involving multiple testing, *P*-value adjustment was performed using method-appropriate procedures. All interpretations were grounded in the hierarchical structure of the analytical workflow and aligned with the overarching biological hypothesis.

The data preprocessing and analysis were all conducted using the R software (Version 4.4.0). The independent Student’s t-test was used to assess the statistical significance of continuous variables with normal distribution unless specified otherwise. The Wilcoxon rank-sum test was used to evaluate the variables without normal distribution. Spearman correlation analysis was applied to measure correlations between genes. For high-dimensional transcriptomic analyses, multiple testing was controlled using the Benjamini-Hochberg FDR procedure. The primary DEG threshold was adj.P.Val < 0.05. Immune-cell group comparisons and candidate gene-immune cell correlations were also adjusted using BH-FDR. For RT-qPCR validation of the seven primary MFRDEGs, *P* values were calculated for two-group comparisons, and FDR values were calculated using the Benjamini-Hochberg method across the seven tested genes. All the *P* values were two-sided unless stated otherwise, and *P* < 0.05 was considered statistically significant.

## 3 Results

### 3.1 Revised workflow of the bioinformatics analysis

**Fig 1:**
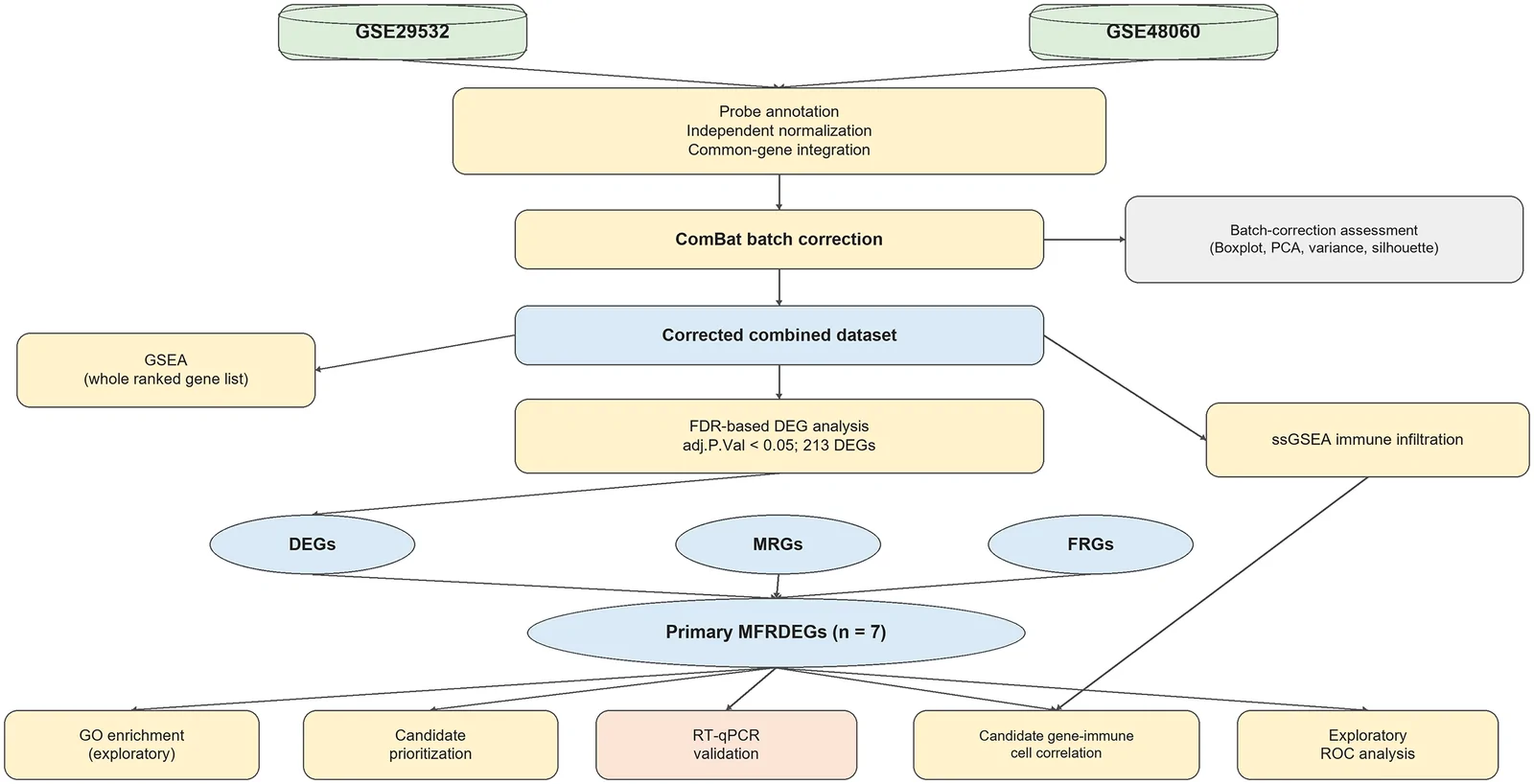
Revised workflow of the bioinformatics analysis. The workflow included dataset collection, probe annotation, normalization, batch correction, FDR-based DEG analysis, MFRDEG identification, GO/GSEA analysis, candidate prioritization, ssGSEA immune infiltration analysis, candidate gene-immune cell correlation analysis, exploratory ROC analysis, and RT-qPCR validation.

The revised analytical workflow is presented in Fig 1. Briefly, peripheral blood transcriptomic datasets GSE29532 and GSE48060 were downloaded from the GEO database, followed by probe annotation, independent normalization, integration based on common genes, and ComBat-based batch correction. Differential expression analysis was then performed using an FDR-controlled threshold. Macrophage- and ferroptosis-related candidate genes were identified by intersecting FDR-supported DEGs with curated MRG and FRG sets. Functional annotation, whole-ranked-gene GSEA, candidate prioritization, ssGSEA immune infiltration analysis, candidate gene-immune cell correlation analysis, exploratory ROC analysis, and RT-qPCR validation of the FDR-supported primary MFRDEGs were subsequently performed.

### 3.2 Data integration and batch-correction quality assessment

**Fig 2:**
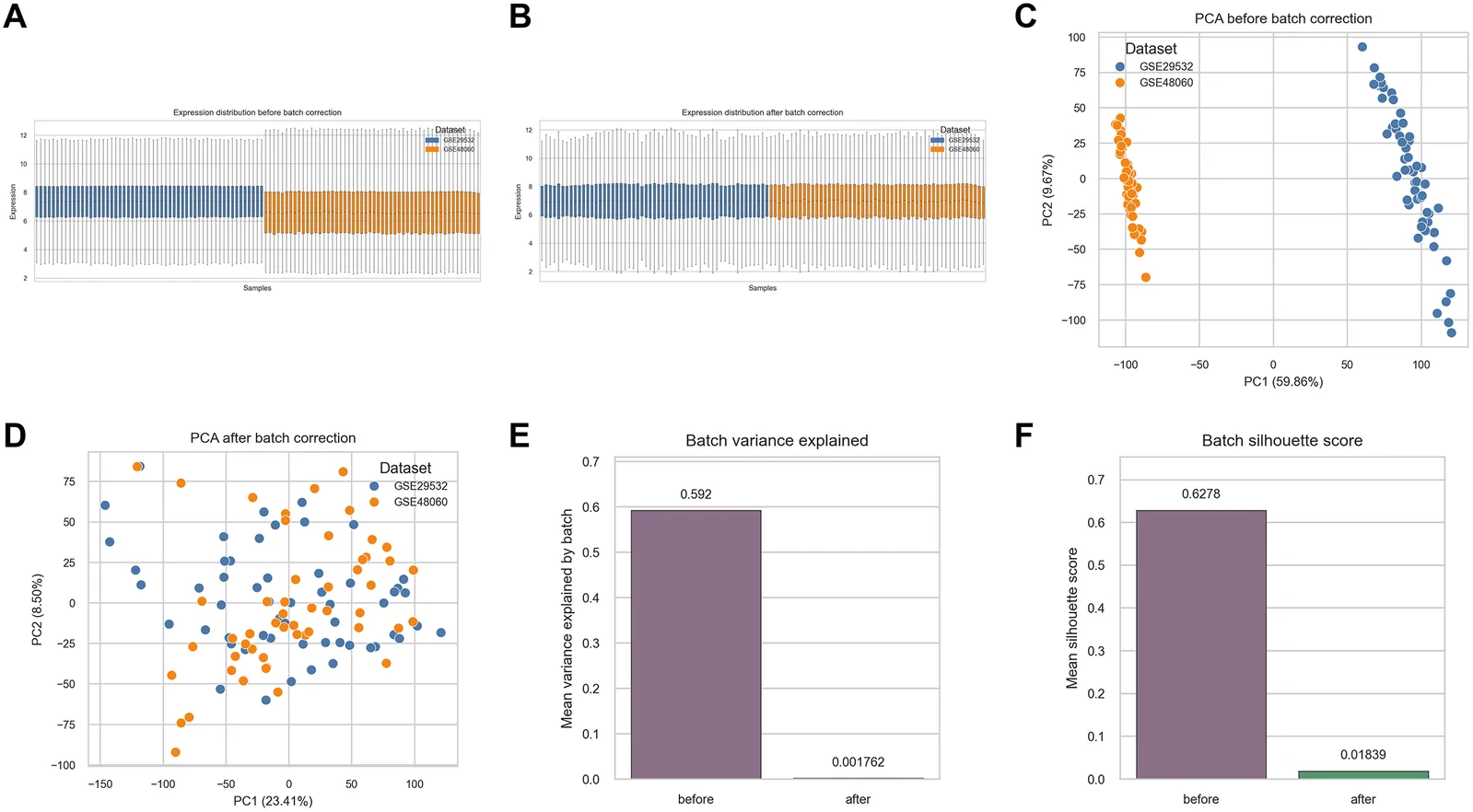
Data preprocessing and batch-correction quality assessment. (A) Boxplot of expression distributions before batch correction. (B) Boxplot of expression distributions after ComBat correction. (C) PCA before batch correction. (D) PCA after batch correction. (E) Mean batch-explained variance before and after correction. (F) Batch silhouette score before and after correction.

After probe-to-gene conversion and dataset-level normalization, the two datasets were integrated using 15,332 shared gene symbols across 107 peripheral blood samples. Because GSE29532 and GSE48060 were generated using different microarray platforms, ComBat correction was applied while retaining disease group information in the model. Before correction, the sample expression distributions and PCA results showed evident platform-associated variation. After correction, the expression distributions became more comparable, and the PCA pattern indicated reduced dataset-driven separation (Fig 2A-D). Quantitative evaluation further supported the effectiveness of batch correction: the mean batch-explained variance decreased from 0.5920 to 0.00176, and the batch silhouette score decreased from 0.6278 to 0.0184 (Fig 2E-F). Extended quality-control plots, including density plots, RLE plots, group silhouette evaluation, and batch-variance distributions, are shown in S1 Fig.

### 3.3 FDR-supported DEGs and primary MFRDEGs

**Fig 3:**
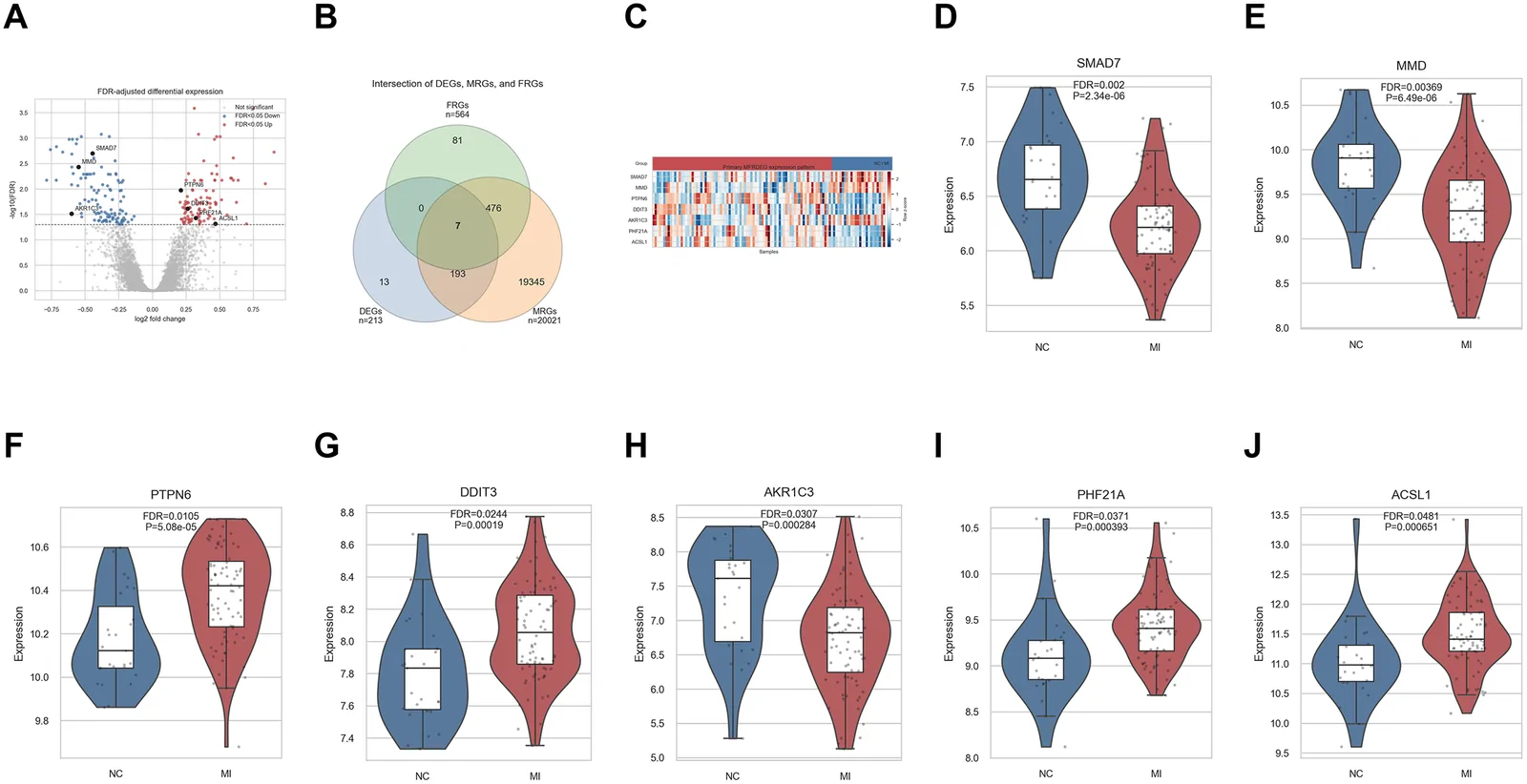
FDR-supported DEGs and primary MFRDEGs. (A) Volcano plot of DEGs between MI and NC samples using adj.P.Val < 0.05. (B) Intersection of FDR-supported DEGs, MRGs, and FRGs. (C) Heatmap of the seven primary MFRDEGs. (D) SMAD7 expression. (E) MMD expression. (F) PTPN6 expression. (G) DDIT3 expression. (H) AKR1C3 expression. (I) PHF21A expression. (J) ACSL1 expression.

Differential expression analysis was performed using limma on the ComBat-corrected expression matrix. Using adj.P.Val < 0.05 as the primary threshold, 213 DEGs were identified between the MI and NC groups, including 101 upregulated genes and 112 downregulated genes **(**Fig 3A). The FDR-supported DEGs were then intersected with MRGs and FRGs to identify macrophage- and ferroptosis-related candidate genes **(**Fig 3B). This intersection yielded seven primary MFRDEGs: SMAD7, MMD, PTPN6, DDIT3, AKR1C3, PHF21A, and ACSL1. The expression pattern of these seven genes across samples is shown in Fig 3C. Individual expression comparisons further demonstrated the group-level expression differences for each primary MFRDEG between MI and NC samples (Fig 3D-J).

To evaluate the robustness of differential-expression and candidate-gene selection, threshold sensitivity analysis was performed using multiple DEG criteria. The old nominal threshold was retained only as an exploratory sensitivity reference and was not used for the primary conclusion. DEG counts, upregulated and downregulated gene counts, and DEG overlap across thresholds are shown in S2 Fig. **A-D**. Additional MFRDEG and gene-set sensitivity analyses showed how different DEG thresholds and MRG/FRG gene-set combinations influenced candidate selection (S3 Fig. **A-D)**.

### 3.4 Exploratory GO annotation of FDR-supported primary MFRDEGs

**Fig 4:**
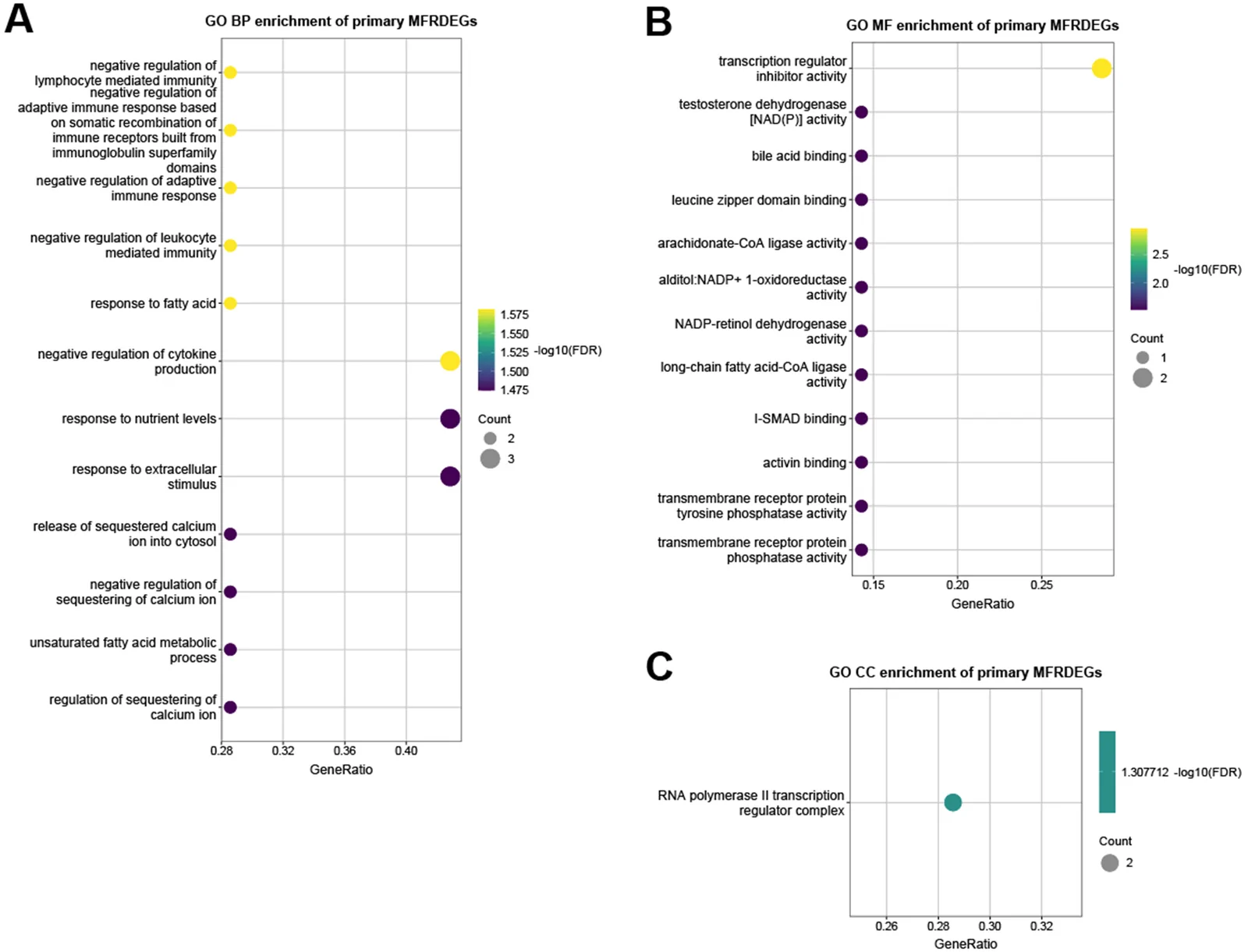
GO enrichment analysis of FDR-supported primary MFRDEGs. (A) GO biological process enrichment. (B) GO molecular function enrichment. (C) GO cellular component enrichment. GO enrichment was performed using clusterProfiler with the org.Hs.e.g.,db default GO-annotated background and BH correction. Because the input set contained only seven FDR-supported primary MFRDEGs, the results were interpreted as exploratory functional annotations.

GO enrichment analysis was performed using the seven FDR-supported primary MFRDEGs. The enriched biological process terms were mainly related to immune regulation, leukocyte-mediated immunity, cytokine production, fatty acid response, nutrient-level response, and extracellular stimulus response (Fig 4A and Table 2). Molecular function terms included transcription regulator inhibitor activity and several metabolism-related binding or enzyme activity annotations (Fig 4B and Table 2). The cellular component terms were mainly related to the RNA polymerase II transcription regulator complex (Fig 4C and Table 2). Because only seven genes were used as input and the default org.Hs.e.g.,db GO-annotated background was applied, these findings were interpreted as exploratory functional annotations rather than robust pathway-level mechanistic evidence. Primary KEGG enrichment did not yield stable significant results and was therefore not emphasized as a main result.

**Table 2:** GO enrichment results for FDR-supported primary MFRDEGs.

ONTOLOGY	ID	Description	GeneRatio	BgRatio	pvalue	p.adjust	qvalue
BP	GO:0002707	negative regulation of lymphocyte mediated immunity	2/7	45/18862	1.16e-04	0.02614	0.01075
BP	GO:0002823	negative regulation of adaptive immune response based on somatic recombination of immune receptors built from immunoglobulin superfamily domains	2/7	47/18862	1.27e-04	0.02614	0.01075
BP	GO:0002820	negative regulation of adaptive immune response	2/7	52/18862	1.55e-04	0.02614	0.01075
BP	GO:0002704	negative regulation of leukocyte mediated immunity	2/7	53/18862	1.61e-04	0.02614	0.01075
BP	GO:0070542	response to fatty acid	2/7	61/18862	2.14e-04	0.02614	0.01075
BP	GO:0001818	negative regulation of cytokine production	3/7	367/18862	2.41e-04	0.02614	0.01075
BP	GO:0031667	response to nutrient levels	3/7	451/18862	4.42e-04	0.03354	0.01379
BP	GO:0009991	response to extracellular stimulus	3/7	477/18862	5.21e-04	0.03354	0.01379
MF	GO:0140416	transcription regulator inhibitor activity	2/7	18/18337	1.91e-05	0.001124	3.01e-04
MF	GO:0030283	testosterone dehydrogenase [NAD(P)] activity	1/7	10/18337	0.003812	0.02883	0.007717
MF	GO:0032052	bile acid binding	1/7	11/18337	0.004192	0.02883	0.007717
MF	GO:0043522	leucine zipper domain binding	1/7	11/18337	0.004192	0.02883	0.007717
MF	GO:0047676	arachidonate-CoA ligase activity	1/7	11/18337	0.004192	0.02883	0.007717
MF	GO:0004032	alditol:NADP+ 1-oxidoreductase activity	1/7	12/18337	0.004573	0.02883	0.007717
CC	GO:0090575	RNA polymerase II transcription regulator complex	2/7	170/19520	0.001539	0.04924	0.02753

Note: GO, Gene Ontology; BP, Biological Process; CC, Cellular Component; MF, Molecular Function; MFRDEGs, Macrophage-Ferroptosis Related Differentially Expressed Genes.

### 3.5 Whole-ranked-gene GSEA

**Fig 5:**
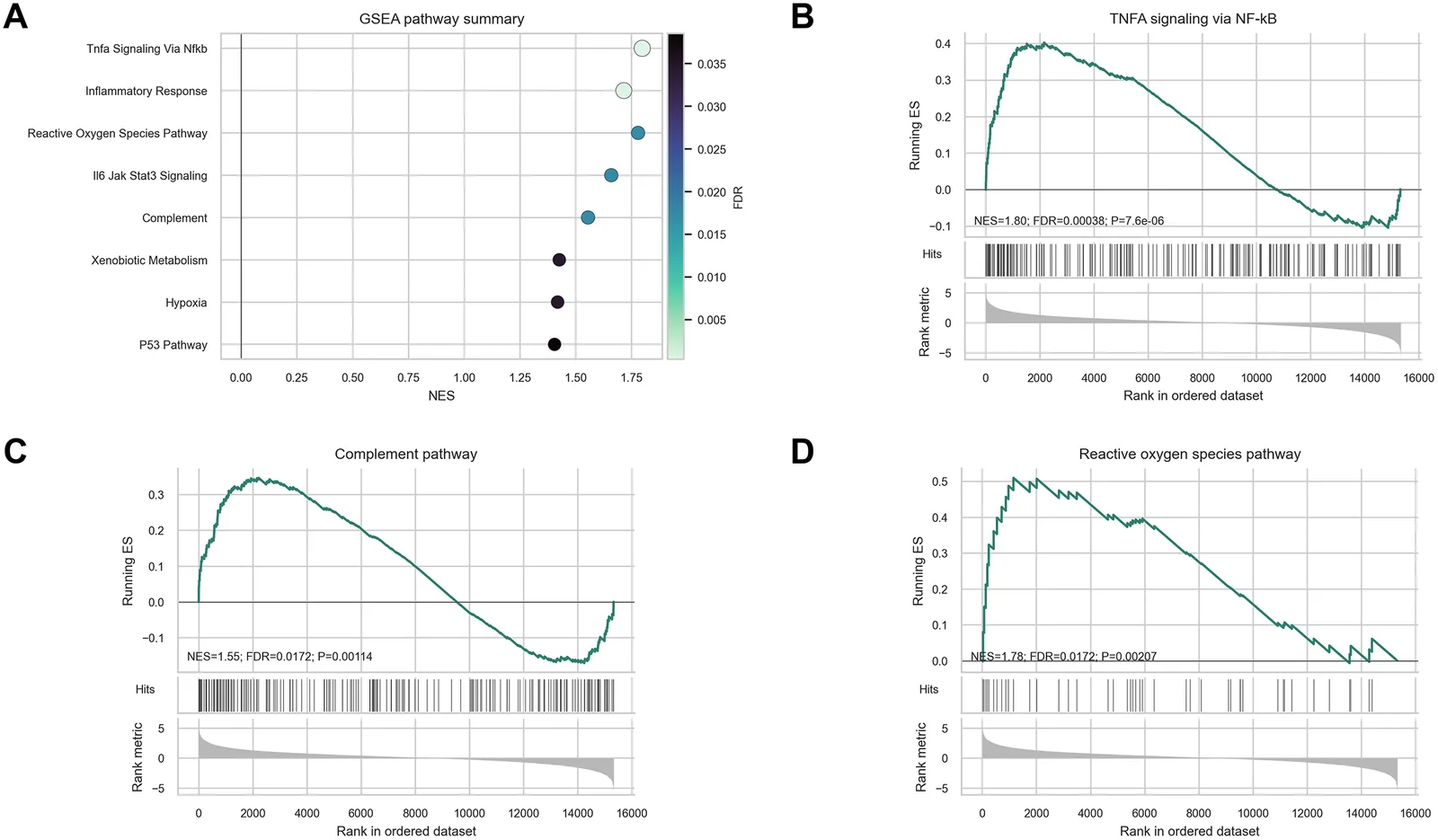
Whole-ranked-gene GSEA. (A) Summary dot plot of FDR-significant Hallmark GSEA pathways. (B) GSEA curve for TNFA signaling via NF-kB. (C) GSEA curve for the Complement pathway. (D) GSEA curve for the reactive oxygen species pathway. Each GSEA curve shows one pathway and reports NES and FDR.

To complement the candidate-gene analysis, GSEA was performed using the full ranked gene list rather than a DEG-truncated list. The pathway-level summary showed several FDR-significant transcriptional programs associated with MI (Fig 5A and Table 3). TNFA signaling via NF-kB was enriched in the MI/up-ranked direction, indicating inflammatory transcriptional activation (Fig 5B). The Complement pathway was also enriched and was interpreted as complement-related innate immune signaling rather than a macrophage-specific pathway (Fig 5C). In addition, the reactive oxygen species pathway was enriched, supporting oxidative stress-related transcriptional changes in MI (Fig 5D). These GSEA findings provided pathway-level association evidence but were not interpreted as proof of causal mechanisms.

**Table 3:** Selected GSEA results from the full ranked gene list.

ID	Collection	NES	p value	p adjust	Direction	Interpretation
HALLMARK_TNFA_SIGNALING_VIA_NFKB	MSigDB Hallmark	1.798	7.60e-06	3.80e-04	enriched in MI/up-ranked genes	inflammatory signaling
HALLMARK_COMPLEMENT	MSigDB Hallmark	1.555	0.001139	0.01723	enriched in MI/up-ranked genes	complement-related innate immune signaling
HALLMARK_REACTIVE_OXYGEN_SPECIES_PATHWAY	MSigDB Hallmark	1.779	0.002067	0.01723	enriched in MI/up-ranked genes	oxidative stress-related transcriptional program

Note: GSEA, Gene Set Enrichment Analysis; NES, Normalized Enrichment Score.

### 3.6 Candidate prioritization without interaction-derived hub-gene definition

**Fig 6:**
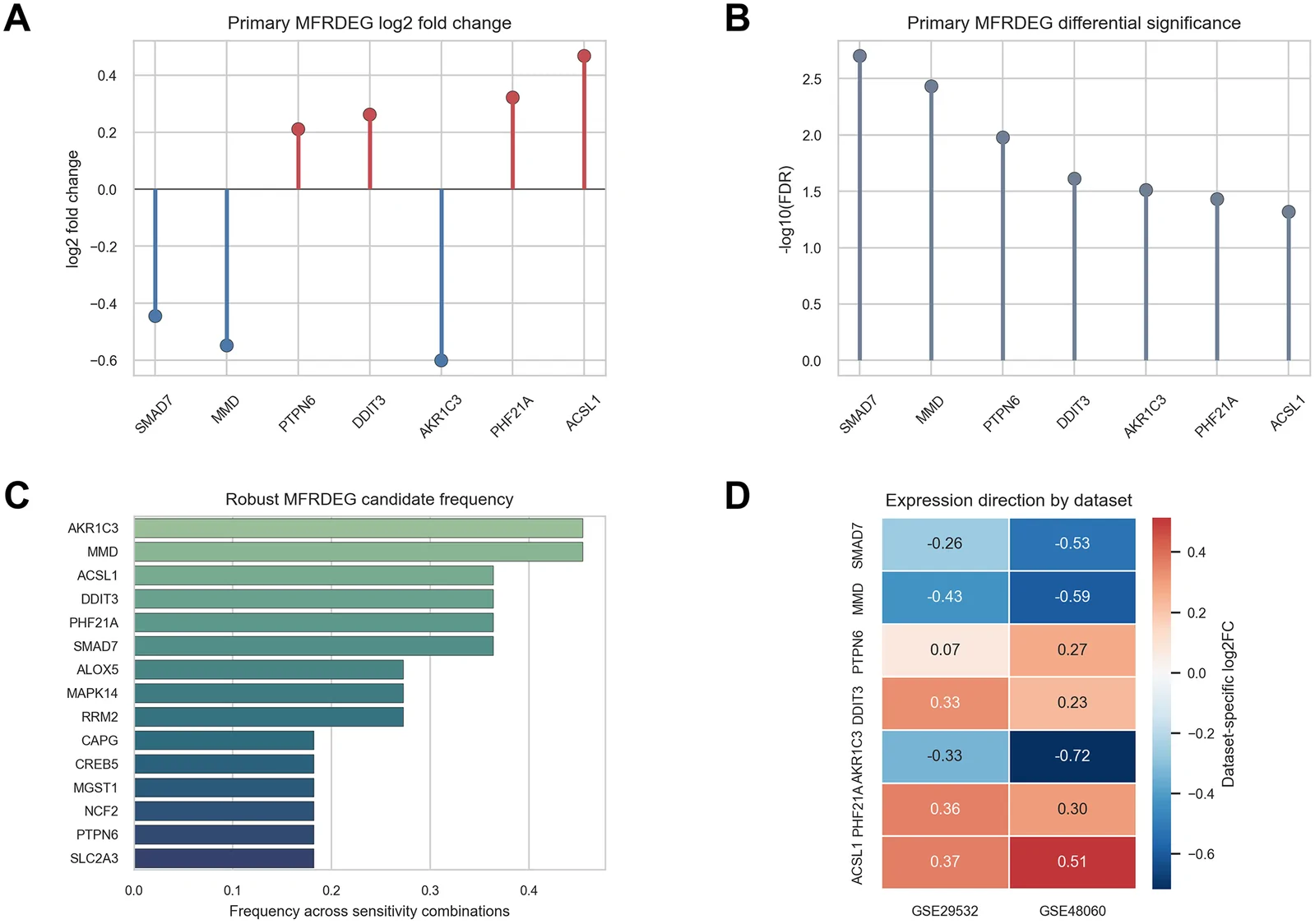
Prioritization of macrophage- and ferroptosis-related candidate genes. (A) log2FC values of the seven primary MFRDEGs. (B) FDR significance of the seven primary MFRDEGs. (C) Robust MFRDEG frequency across sensitivity analyses. (D) Dataset-level expression-direction consistency. This figure presents candidate prioritization evidence rather than network-derived hub-gene selection.

The seven FDR-supported primary MFRDEGs were further evaluated for candidate prioritization. Because the revised PPI analysis did not generate reliable interactions among these seven genes, an interaction-derived hub-gene set was not defined. Instead, candidate prioritization was based on multiple candidate-level features, including log2FC, FDR significance, robustness across sensitivity analyses, dataset-level expression-direction consistency, immune-correlation evidence, and biological relevance (Fig 6A-D). The supplementary candidate evidence matrix further summarized the evidence tier of candidate genes, previous prioritization status, immune-correlation evidence, exploratory ROC availability, and RT-qPCR selection status (S4 Fig).

### 3.7 ssGSEA immune-cell-related signatures

**Fig 7:**
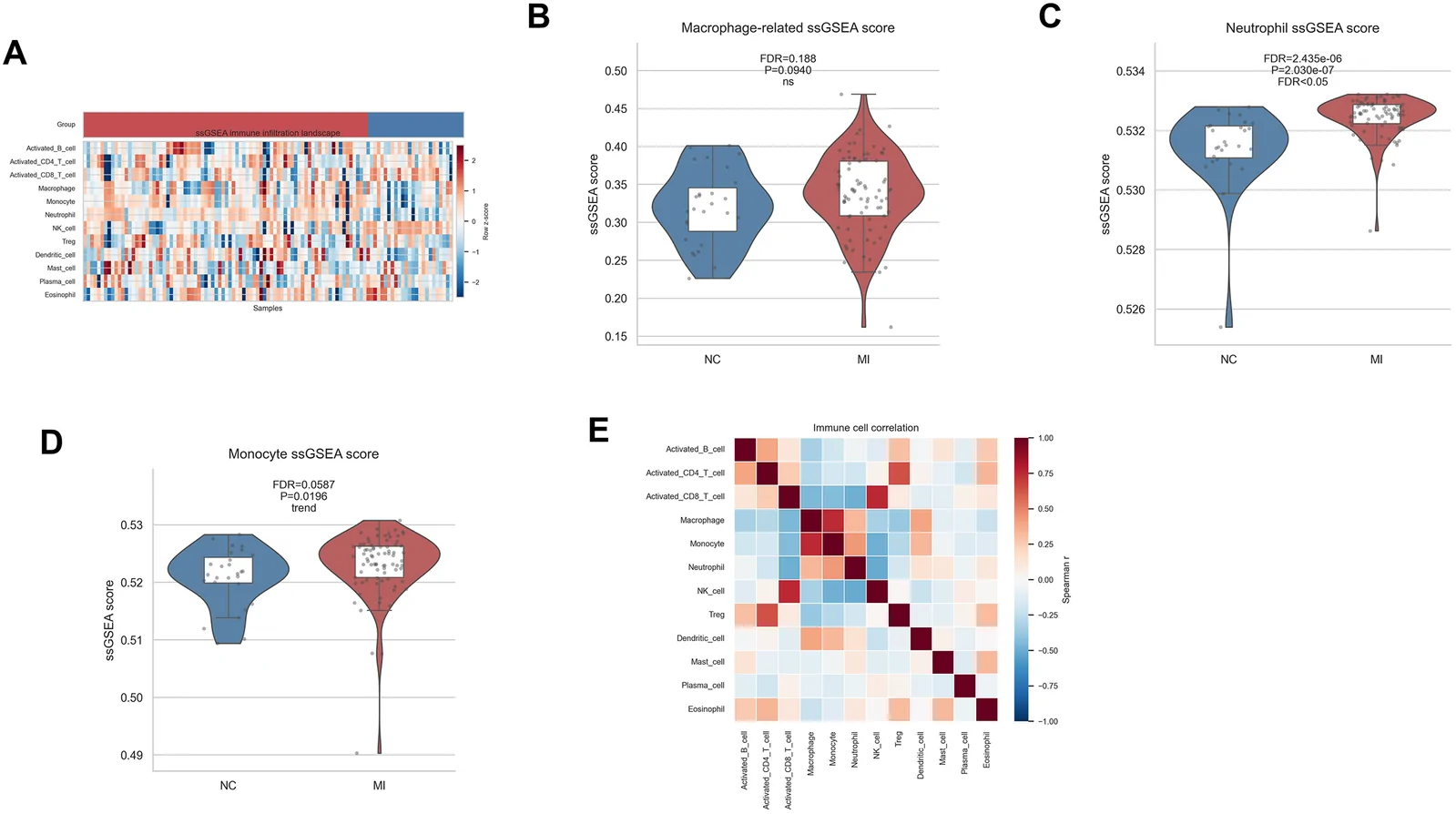
ssGSEA immune-cell-related signatures. (A) Heatmap of ssGSEA-derived immune-cell-related scores. (B) Macrophage-related ssGSEA score. (C) Neutrophil-related ssGSEA score. (D) Monocyte-related ssGSEA score. (E) Spearman correlation heatmap among immune-cell-related scores. *P* values for group comparisons were adjusted using the Benjamini-Hochberg FDR procedure.

Immune-cell-related transcriptional signatures were evaluated using ssGSEA on the batch-corrected expression matrix. The overall immune-score heatmap suggested broad immune-related variation across samples (Fig 7A). Among the immune-cell-related scores highlighted in the main figure, macrophage-related ssGSEA score showed an upward trend in MI but did not remain significant after BH-FDR correction (Fig 7B). Neutrophil-related ssGSEA score was significantly increased in MI after FDR correction (Fig 7C). Monocyte-related ssGSEA score showed a trend-level alteration but did not reach FDR < 0.05 (Fig 7D). Therefore, neutrophil-related alteration represented the most robust immune-cell-related change in this analysis, whereas macrophage- and monocyte-related results were interpreted cautiously. Correlations among immune-cell-related scores are shown in Fig 7E. Full immune-cell comparison results are provided in S5 Fig.

### 3.8 Candidate gene-immune cell correlation analysis

**Fig 8:**
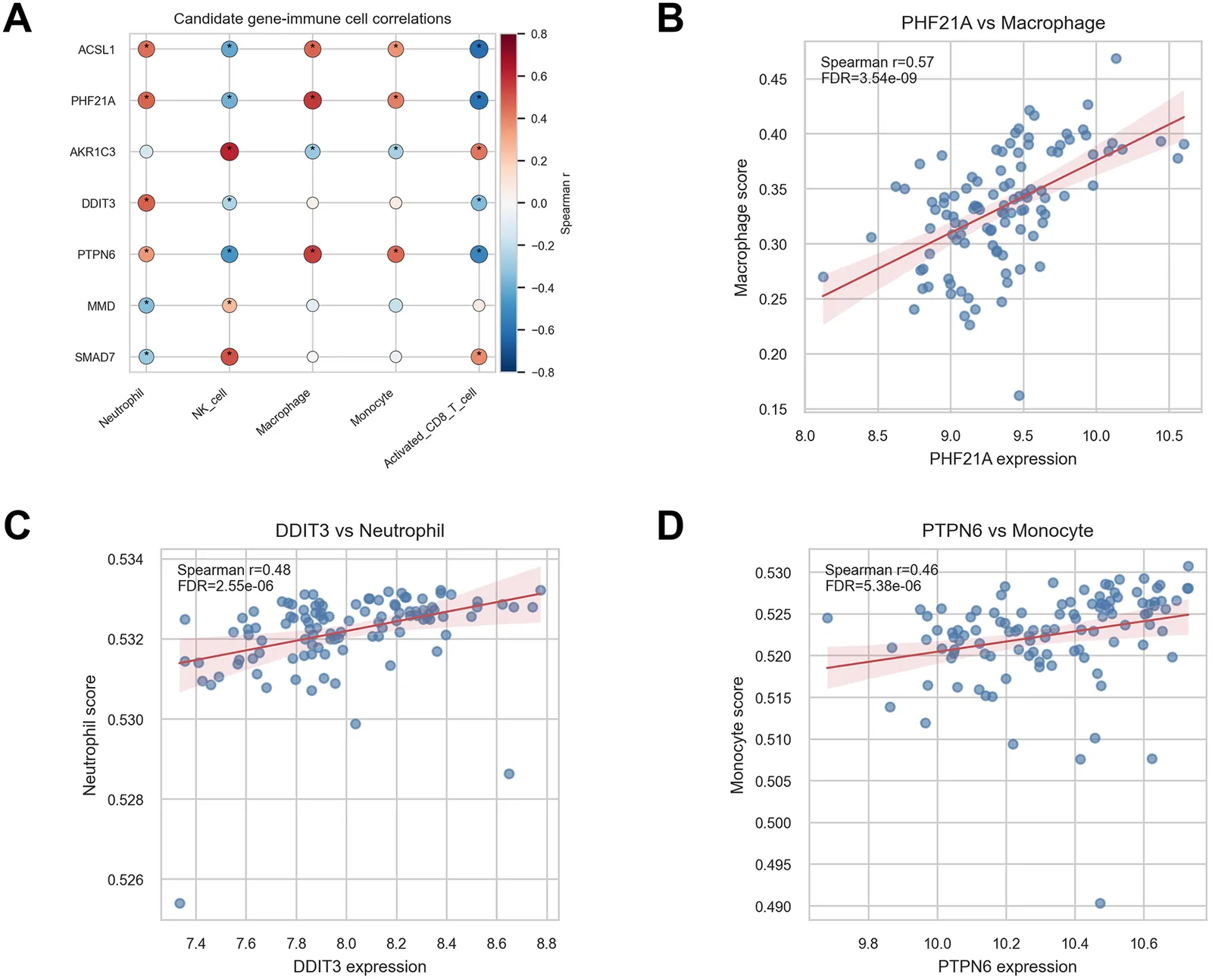
Candidate gene-immune cell correlation analysis. (A) Bubble plot showing Spearman correlations between candidate genes and immune-cell-related scores. (B) Correlation between PHF21A expression and the macrophage-related ssGSEA score. (C) Correlation between DDIT3 expression and the neutrophil-related ssGSEA score. (D) Correlation between PTPN6 expression and the monocyte-related ssGSEA score. *P* values were adjusted using BH-FDR correction.

Spearman correlation analysis was used to evaluate associations between candidate gene expression and ssGSEA-derived immune-cell-related scores. Correlations were interpreted after BH-FDR correction across tested gene-cell pairs. The overall correlation pattern is shown in Fig 8A. Representative candidate gene-immune cell associations were further visualized, including PHF21A with macrophage-related score, DDIT3 with neutrophil-related score, and PTPN6 with monocyte-related score (Fig 8B-D). These results suggested association-level links between candidate genes and immune-cell-related transcriptional signatures but did not prove direct cellular regulation.

### 3.9 Exploratory ROC analysis

**Fig 9:**
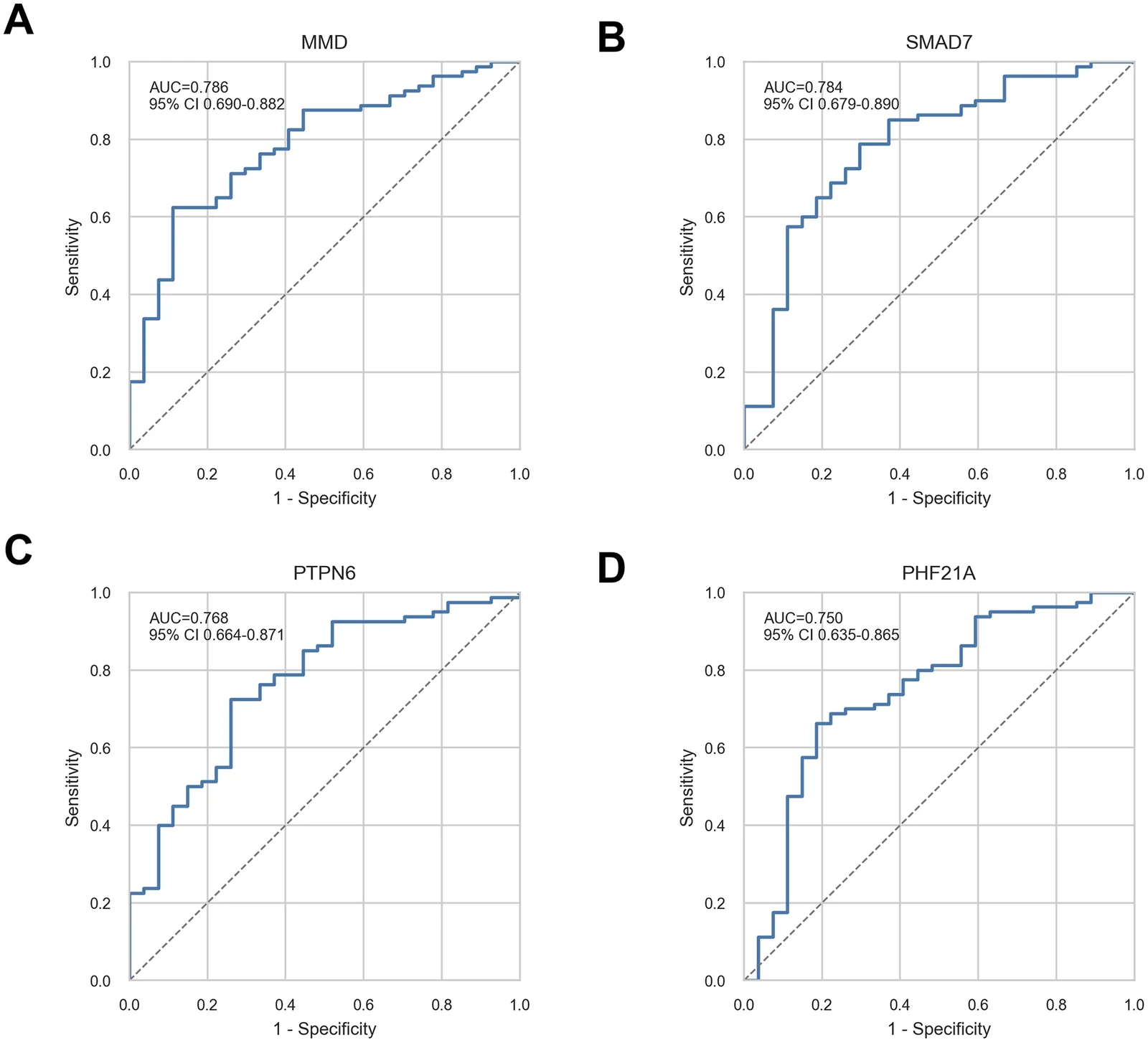
Exploratory ROC analysis of candidate genes. (A) ROC curve for MMD. (B) ROC curve for SMAD7. (C) ROC curve for PTPN6. (D) ROC curve for PHF21A. AUC values indicate exploratory discriminatory performance only and require independent validation.

Exploratory ROC analysis was performed to estimate the discriminatory performance of selected candidate genes. In the main figure, ROC curves were shown for MMD, SMAD7, PTPN6, and PHF21A (Fig 9A-D). These analyses were interpreted only as exploratory performance estimates and not as validated diagnostic models. Full ROC/AUC summaries and additional gene-specific ROC curves are provided in S6 Fig.

### 3.10 RT-qPCR validation of FDR-supported primary MFRDEGs

**Fig 10:**
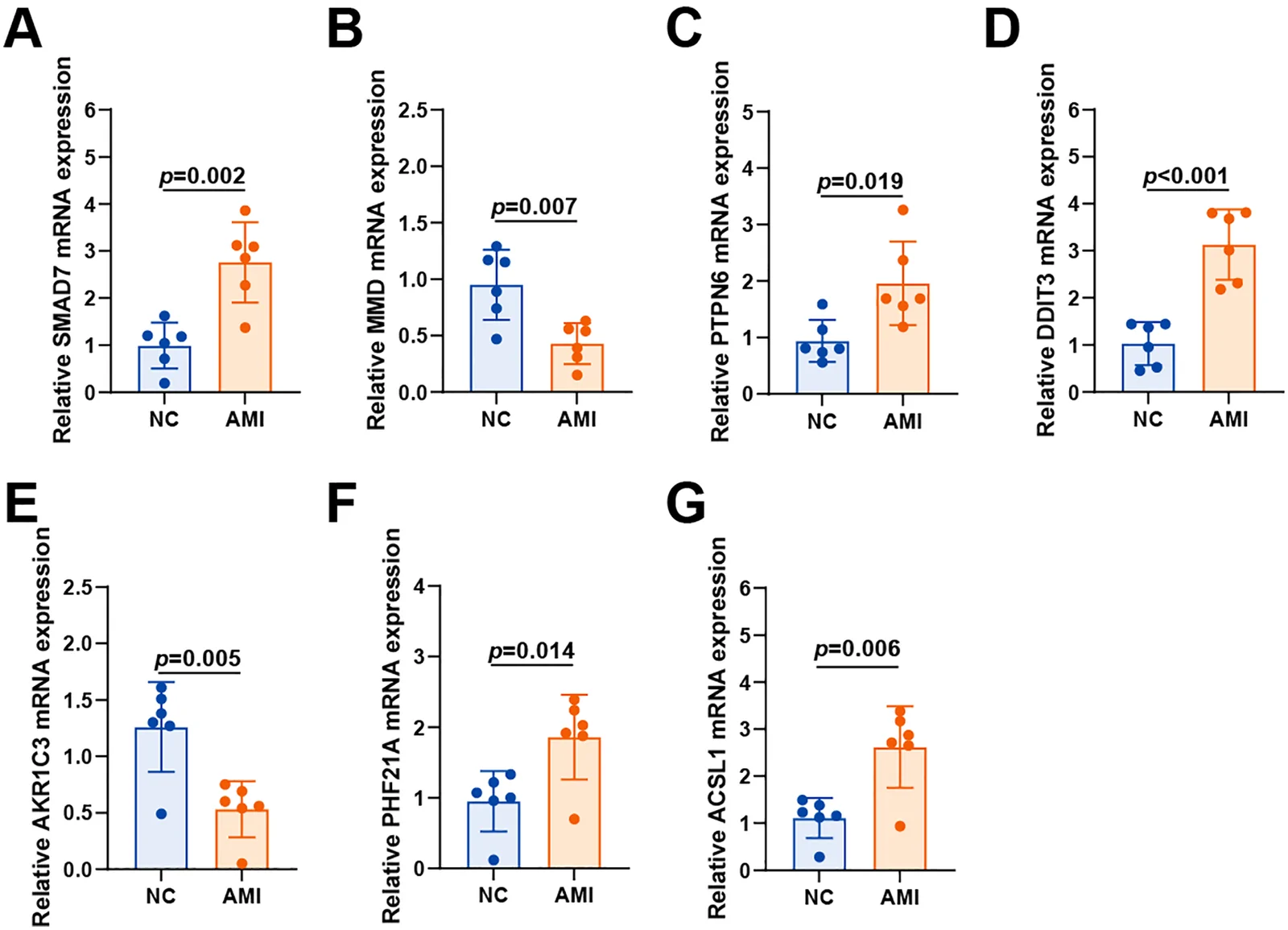
RT-qPCR validation of FDR-supported primary MFRDEGs in peripheral blood samples. (A) SMAD7 mRNA expression. (B) MMD mRNA expression. (C) PTPN6 mRNA expression. (D) DDIT3 mRNA expression. (E) AKR1C3 mRNA expression. (F) PHF21A mRNA expression. (G) ACSL1 mRNA expression. Relative mRNA expression was measured by RT-qPCR in NC and AMI peripheral blood samples. Each point represents one sample. P values were calculated for two-group comparisons, and FDR values were calculated using the Benjamini-Hochberg method across the seven tested genes. Data are shown as mean ± SD.

To further validate the expression patterns of the FDR-supported primary MFRDEGs in independent peripheral blood samples, RT-qPCR was performed for SMAD7, MMD, PTPN6, DDIT3, AKR1C3, PHF21A, and ACSL1. Compared with the NC group, SMAD7 expression was significantly increased in the AMI group (Fig 10A). MMD expression was significantly decreased in the AMI group (Fig 10B). PTPN6 expression was significantly increased in the AMI group (Fig 10C). DDIT3 expression was also significantly increased (Fig 10D). AKR1C3 expression was significantly decreased in the AMI group (Fig 10E). PHF21A expression was significantly increased (Fig 10F), and ACSL1 expression was significantly increased in the AMI group (Fig 10G). The direction of RT-qPCR validation was consistent with the transcriptomic analysis for all seven primary MFRDEGs. These results provided preliminary experimental support for the revised FDR-supported candidate-gene findings.

## 4 Discussion

During the inflammatory response and tissue repair post-MI, the dynamic alteration of macrophages is recognized as a critical factor affecting myocardial remodeling [24]. Concurrently, the association between ferroptosis and myocardial injury as well as adverse ventricular remodeling following AMI has also received sustained attention [5]. Against this research background, the present study screened candidate molecules based on peripheral blood transcriptome data through the intersection of MRGs and FRGs, and conducted an integrated assessment of their potential biological significance in MI by combining immune infiltration analysis and RT-qPCR validation. Compared with the previous exploratory workflow, the revised analysis adopted FDR-controlled differential expression as the primary criterion and identified seven FDR-supported primary MFRDEGs: SMAD7, MMD, PTPN6, DDIT3, AKR1C3, PHF21A, and ACSL1. These results provide candidate-level evidence for macrophage- and ferroptosis-related transcriptional alterations in MI rather than definitive causal mechanisms.

This study employed an integrated bioinformatics approach to identify 213 FDR-supported DEGs from the consolidated dataset, including 101 upregulated and 112 downregulated genes. Subsequently, seven MFRDEGs were systematically screened. Among them, SMAD7, PTPN6, DDIT3, PHF21A, and ACSL1 were increased in the AMI group in the RT-qPCR validation cohort, whereas MMD and AKR1C3 were decreased. The direction of RT-qPCR validation was consistent with the transcriptomic analysis for all seven primary MFRDEGs, providing preliminary experimental support for the revised candidate-gene findings.

Previous studies have depicted that Smad7 could suppress TGF-β/activin-like signaling, predominantly inducing anti-inflammatory effects in macrophages [25,26]. In the ischemia/reperfusion model, atorvastatin elevated SMAD7 expression, inhibited cardiac hepcidin expression, and increased ferroportin 1 in cardiomyocytes. Subsequently, the myocardial iron efflux was enhanced, thereby alleviating ferroptosis in cardiomyocytes [27]. Recent studies have demonstrated that *SMAD7* is involved in modulation of myocardial fibroblast activity following MI via the TGF-β and ErbB2 signaling pathways, thereby conferring protection against post-infarction heart failure [28]. Therefore, the identification and RT-qPCR validation of SMAD7 in this study support its potential relevance as a macrophage- and ferroptosis-related candidate gene in MI, although its specific role requires further mechanistic verification.

A clinical cohort study has shown that the up-regulation *ACSL1* gene could be a diagnostic molecular marker for AMI [29]. A previous study has demonstrated that *ACSL1* is up-regulated in AMI patients, and the up-regulation promotes ferroptosis in cardiomyocytes, as demonstrated in cellular experiments [30]. Furthermore, evidence indicates that *TLR4* activation in macrophages upregulates *ACSL1* expression [31]. Moreover, the ablation of *ACSL1* attenuates diabetic atherosclerosis in murine models, suggesting a functional role for *ACSL1* in macrophage lipotoxicity [32]. In the revised analysis, ACSL1 was retained as an FDR-supported primary MFRDEG and was significantly increased in AMI peripheral blood by RT-qPCR, supporting its candidacy at the interface of lipid metabolism, macrophage activation, and ferroptosis-related stress.

DDIT3, also known as CHOP, is a downstream component of the endoplasmic reticulum stress pathway. DDIT3 protein overexpression has been associated with the induction of cell death [33]. Accumulating evidence indicates that macrophage phenotype switching may play a key regulatory role in inflammation, myocardial injury, and cardiac repair [34,35]. A recent study has identified DDIT3 as an immune-related ferroptosis gene differentially expressed in acute MI, depicting its diagnostic and therapeutic target potential [36]. Consistent with these studies, DDIT3 was identified as an FDR-supported primary MFRDEG in the present analysis and was significantly increased in AMI samples by RT-qPCR. These findings suggest that DDIT3 may represent a stress-response-related candidate linking immune alteration and ferroptosis-related injury in MI, but functional experiments are required to define its specific cellular source and biological role.

MMD, PTPN6, AKR1C3, and PHF21A were also identified as FDR-supported primary MFRDEGs and validated by RT-qPCR. MMD and AKR1C3 were decreased in AMI samples, whereas PTPN6 and PHF21A were increased. Compared with SMAD7, ACSL1, and DDIT3, these genes have been less extensively characterized in the specific context of MI-related macrophage-ferroptosis interactions. Therefore, we interpret them conservatively as computationally prioritized and experimentally supported candidate genes that require further validation in independent cohorts and mechanistic assays.

GO enrichment analysis revealed that the seven primary MFRDEGs were associated with immune regulation, fatty-acid response, cytokine production, transcriptional regulation, and cellular stress-related annotations. Fatty acid metabolism is critical for energy production and cell signaling and is pivotal for preserving cardiac function while responding to ischemic injury [37]. PPARα has been reported to be involved in transcriptional regulation of genes in the fatty acid utilization pathway [38]. Previous studies have also explored adipose-derived or adipocyte-related biology in myocardial repair after infarction [39,40]. However, because the GO analysis in this study was based on only seven input genes and used the default org.Hs.e.g.,db GO-annotated background, the GO findings should be interpreted as exploratory functional annotations rather than definitive pathway-level evidence. Primary KEGG enrichment did not yield robust significant terms in the revised analysis, and previous KEGG-centered interpretations were therefore not retained as main conclusions.

The GSEA results provided complementary information at the whole-ranked-gene level. Instead of relying on a DEG-truncated list, the revised GSEA identified TNFA/NF-kB inflammatory signaling, complement-related innate immune signaling, and reactive oxygen species-related transcriptional programs enriched in the MI/up-ranked direction. These pathways are consistent with inflammatory activation and oxidative stress in MI. Nevertheless, GSEA findings represent transcriptomic pathway associations and should not be interpreted as direct mechanistic proof.

The immune feature analysis of this study suggests that peripheral immune alterations associated with MI involve innate immune activation. After FDR correction, neutrophil-related ssGSEA scores showed the most robust alteration in MI, whereas macrophage-related and monocyte-related scores showed trend-level changes that did not reach FDR significance. Therefore, macrophage and monocyte signatures should not be described as significantly increased in the revised manuscript. In addition, candidate gene-immune cell correlation analysis provided association-level evidence linking selected MFRDEGs with immune-cell-related transcriptional signatures. Adaptive immune remodeling may also be relevant after MI, as T cell subsets have been implicated in post-MI inflammatory regulation, tissue repair, and cardiac remodeling [41,42]. However, these findings are based on bulk peripheral blood data and cannot resolve tissue-localized immune-cell composition or direct cell-cell communication.

Exploratory ROC analysis was retained to estimate the discriminatory performance of candidate genes, but these results should not be interpreted as validated clinical diagnostic models. The updated RT-qPCR validation of SMAD7, MMD, PTPN6, DDIT3, AKR1C3, PHF21A, and ACSL1 further supported the expression patterns of the FDR-supported primary MFRDEGs. This updated validation strategy directly aligns the experimental validation with the revised candidate-gene screening results and reduces the inconsistency caused by the previously selected qPCR genes.

Although this study systematically integrated multiple public datasets for bioinformatics analysis and further validated the revised primary MFRDEGs by RT-qPCR, certain limitations warrant careful interpretation. First, the current data analysis predominantly reflects the expression profiles of peripheral blood cells and may not fully capture molecular alterations in infarcted myocardial tissue. Second, the collection of MRGs and FRGs used in this study was compiled from multiple databases and literature resources. Despite curation, residual annotation inconsistencies and selection biases remain unavoidable. Third, GO enrichment was performed using a small seven-gene input set and the default org.Hs.e.g.,db background, so it was treated as exploratory annotation. Fourth, the revised PPI analysis did not support a reliable interaction-derived hub-gene model. Finally, although RT-qPCR provided preliminary validation of the seven primary MFRDEGs, larger clinical cohorts, myocardial tissue datasets, and functional experiments are still needed to evaluate their biological roles in MI.

## 5 Conclusion

Using an FDR-controlled reanalysis of peripheral blood transcriptomic datasets, this study identified seven macrophage- and ferroptosis-related candidate genes associated with MI: SMAD7, MMD, PTPN6, DDIT3, AKR1C3, PHF21A, and ACSL1. Integrated GO annotation, whole-ranked-gene GSEA, immune-cell-related ssGSEA analysis, candidate gene-immune cell correlation analysis, exploratory ROC analysis, and RT-qPCR validation collectively supported these genes as candidate-level molecular signals related to immune and stress-response alterations in MI. These findings should be interpreted as hypothesis-generating evidence and require further validation in larger independent cohorts, myocardial tissue datasets, and functional experimental models.

## Supporting information

S1 Fig Extended batch-correction quality control. (A) Density plot before batch correction. (B) Density plot after batch correction. (C) RLE plot before batch correction. (D) RLE plot after batch correction. (E) Group silhouette score before and after correction. (F) Batch variance distribution before correction. (G) Batch variance distribution after correction.
(TIFF)

S2 Fig DEG threshold sensitivity analysis. (A) Total DEG counts across thresholds. (B) Upregulated DEG counts across thresholds. (C) Downregulated DEG counts across thresholds. (D) DEG overlap across thresholds.
(TIFF)

S3 Fig MFRDEG and gene-set sensitivity analysis. (A) MFRDEG counts across DEG thresholds. (B) MFRDEG counts across MRG/FRG gene-set combinations. (C) Robust MFRDEG frequency. (D) Overlap between primary MFRDEGs and robust candidates.
(TIFF)

S4 Fig Candidate evidence matrix. (A) Evidence matrix summarizing candidate-gene evidence, including primary status, robustness, previous prioritization, immune-correlation evidence, exploratory ROC availability, RT-qPCR selection, and final evidence tier. (B) Evidence-tier summary.
(TIFF)

S5 Fig Full immune-cell comparison. (A) Dot plot of FDR results for all immune-cell-related ssGSEA scores. (B) Individual FDR-supported immune-cell comparison not displayed in the main figures.
(TIFF)

S6 Fig Full exploratory ROC/AUC results. (A) AUC values for candidate genes. (B-K) Additional panels show gene-specific exploratory ROC curves where available. All ROC analyses were interpreted as exploratory.
(TIFF)

S1 Table Primer sequence. (XLSX)

## Abbreviations

MI: myocardial infarction
MFRDEG: macrophage and ferroptosis related differentially expressed genes
GEO: gene expression omnibus
NC: normal control
DEG: differentially expressed genes
GO: gene ontology
KEGG: kyoto encyclopedia of genes and genomes
BP: biological process
CC: cellular component
MF: molecular function
PPI: protein-protein interaction
TF: transcription factor
miRNA: microRNA
ROC: receiver operating characteristic
ssGSEA: single-sample gene-set enrichment analysis
PCI: percutaneous coronary intervention
MRG: macrophage related genes
FRG: ferroptosis related genes
PCA: principal component analysis
MCC: maximal clique centrality
MNC: maximum neighborhood component
EPC: edge percolated component
GSEA: gene set enrichment analysis
NES: normalized enrichment score
RT-qPCR: reverse transcription quantitative real-time polymerase chain reaction
NSTEMI: non-ST-segment elevation myocardial infarction
AUC: area under the curve
